# mTOR inhibitors as potential therapeutics for endometriosis: a narrative review

**DOI:** 10.1093/molehr/gaae041

**Published:** 2024-11-23

**Authors:** Akiko Nakamura, Yuji Tanaka, Tsukuru Amano, Akie Takebayashi, Akimasa Takahashi, Tetsuro Hanada, Shunichiro Tsuji, Takashi Murakami

**Affiliations:** Department of Obstetrics and Gynaecology, Shiga University of Medical Science, Otsu, Shiga, Japan; Department of Obstetrics and Gynaecology, Shiga University of Medical Science, Otsu, Shiga, Japan; Department of Obstetrics and Gynaecology, Shiga University of Medical Science, Otsu, Shiga, Japan; Department of Obstetrics and Gynaecology, Shiga University of Medical Science, Otsu, Shiga, Japan; Department of Obstetrics and Gynaecology, Shiga University of Medical Science, Otsu, Shiga, Japan; Department of Obstetrics and Gynaecology, Shiga University of Medical Science, Otsu, Shiga, Japan; Department of Obstetrics and Gynaecology, Shiga University of Medical Science, Otsu, Shiga, Japan; Department of Obstetrics and Gynaecology, Shiga University of Medical Science, Otsu, Shiga, Japan

**Keywords:** mTOR inhibitor, endometriosis, adenomyosis, hormonal resistance, fertility preservation, rapamycin, sirolimus, everolimus

## Abstract

Mammalian target of rapamycin (mTOR) inhibitors have been used clinically as anticancer and immunosuppressive agents for over 20 years, demonstrating their safety after long-term administration. These inhibitors exhibit various effects, including inhibition of cell proliferation, interaction with the oestrogen and progesterone pathways, immunosuppression, regulation of angiogenesis, and control of autophagy. We evaluated the potential of mTOR inhibitors as therapeutic agents for endometriosis, examined the secondary benefits related to reproductive function, and assessed how their side effects can be managed. We conducted a thorough review of publications on the role of the mTOR pathway and the effectiveness of mTOR inhibitors in endometriosis patients. These results indicate that the mTOR pathway is activated in endometriosis. Additionally, mTOR inhibitors have shown efficacy as monotherapies for endometriosis. They may alleviate resistance to hormonal therapy in endometriosis, suggesting a potential synergistic effect when used in combination with hormonal therapy. The potential reproductive benefits of mTOR inhibitors include decreased miscarriage rates, improved implantation, and prevention of age-related follicular loss and ovarian hyperstimulation syndrome. Activation of the mTOR pathway has also been implicated in the malignant transformation of endometriosis. Preclinical studies suggest that the dosage of mTOR inhibitors needed for treating endometriosis may be lower than that required for anticancer or immunosuppressive therapy, potentially reducing dosage-dependent side effects. In conclusion, while mTOR inhibitors, which allow for pregnancy during oral administration, show potential for clinical use in all stages of endometriosis, current evidence is limited to preclinical studies, and further research is needed to confirm clinical effectiveness.

## Graphical Abstract

**Graphical Abstract:**
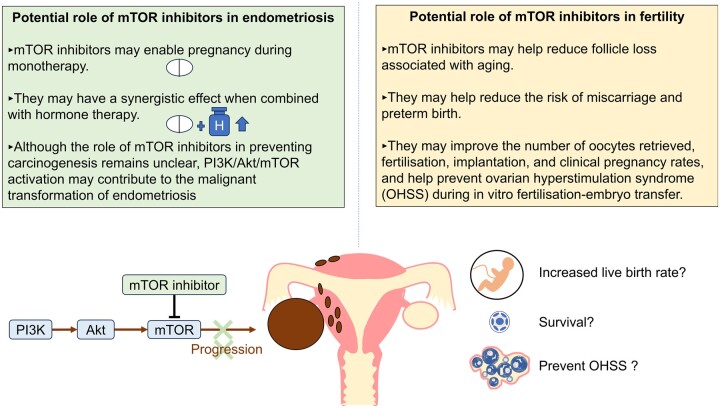
**Potential role of mTOR inhibitors in endometriosis and fertility.** mTOR, mammalian target of rapamycin; PI3K, phosphatidylinositol 3-kinase; Akt, protein kinase B; OHSS, ovarian hyperstimulation syndrome.

## Introduction

Endometriosis is a common gynaecological condition characterized by dysmenorrhoea, chronic pelvic pain, infertility, and an increased risk of malignancy. While endocrine therapy remains the cornerstone of medical management, a subset of patients are unable to continue treatment due to adverse effects, and others develop resistance to endocrine therapy (Li *et al.*, 2020). Furthermore, one of the inherent limitations of endocrine therapy is the requirement for contraception during treatment, which poses a challenge for patients desiring pregnancy. Consequently, there is a growing need to develop non-endocrine-active therapeutic options for endometriosis, either as standalone treatments or in conjunction with hormone therapy.

The mammalian target of rapamycin (mTOR) pathway functions as a protein kinase that regulates metabolism, catabolism, the immune response, autophagy, survival, and proliferation. This pathway is modulated by the upstream regulators phosphatidylinositol 3-kinase (PI3K) and protein kinase B (Akt). The mTOR pathway consists of mTOR Complex 1 (mTORC1) and mTOR Complex 2, and dysregulation of mTORC1 is linked to ageing, cancer, metabolic syndrome, inflammatory diseases, and various other conditions (Zou *et al.*, 2020; Panwar *et al.*, 2023).

Several recent reviews have examined the relationship between endometriosis and the PI3K/Akt/mTOR pathway. For instance, comprehensive reviews on the signalling pathways involved in endometriosis have discussed the potential role of PI3K/Akt/mTOR (Assaf *et al.*, 2022; Driva *et al.*, 2022; Zhang *et al.*, 2023b; Adilbayeva and Kunz, 2024; Wang *et al.*, 2024). Additionally, mTOR signalling has been highlighted in reviews focusing on endometriosis-associated malignancies (Gadducci and Zannoni, 2020; Samartzis *et al.*, 2020; Bartiromo *et al.*, 2022; Driva *et al.*, 2023; Centini *et al.*, 2024; Hablase *et al.*, 2024; Steinbuch et al., 2024). Some reviews have also considered PI3K/Akt/mTOR inhibitors as potential candidates for therapies targeting specific signalling pathways, immunomodulatory approaches, and autophagy in endometriosis (Hung *et al.*, 2021; Samare-Najaf *et al.*, 2023; Zhang *et al.*, 2023b; Kobayashi *et al.*, 2024).

Despite the growing interest in the PI3K/Akt/mTOR signalling pathway in endometriosis, there is still a significant gap in understanding the role and toxicity management of mTOR inhibitors, the only class of PI3K/Akt/mTOR pathway inhibitors with a long history of clinical use. This gap persists despite extensive preclinical studies addressing its efficacy. Notably, essential information relevant to the use of mTOR inhibitors in endometriosis patients is lacking, including their potential to improve hormone resistance in oestrogen-dependent diseases (e.g. breast and endometrial cancers), their application as immunosuppressants, their role in enhancing fertility in women seeking pregnancy, and effective toxicity management in clinical settings.

This study aimed to review systematically the current evidence on the efficacy and feasibility of mTOR inhibitors in the treatment of endometriosis, specifically addressing the critical gaps identified in the existing literature.

## Methods

The process of article selection and inclusion criteria involved searching the PubMed database for articles published up to October 2024 using the keywords ‘endometriosis’ and ‘mTOR’ or ‘rapamycin’. Additionally, we searched the PubMed database for clinical trials on mTOR inhibitors, their toxicity (including teratogenicity), and the relationship between mTOR inhibitors and fertility.

The initial search yielded 122 articles, which were screened by reviewing their titles and abstracts to assess their relevance to the topic. Potentially relevant articles were selected for full-text review. To meet the inclusion criteria, studies had to be published in peer-reviewed academic journals written in English and specifically address either the involvement of the mTOR pathway in endometriosis or the inhibition of this pathway. The identified articles and related studies were then synthesized to provide an overview of the dynamics of the mTOR pathway in endometriosis and the feasibility of using mTOR inhibitors as therapeutic strategies for treating this condition.

## Nature and pathogenesis of endometriosis

Endometriosis is characterized by the abnormal presence of endometrial tissue outside the uterus. Based on the location of the lesions, they are classified into three main subtypes: superficial endometriosis, deep infiltrating endometriosis, and ovarian endometrioma (commonly referred to as a chocolate cyst). The prevalence of endometriosis is high, affecting ∼10% of women of reproductive age. Among patients with endometriosis, 30–50% experience infertility and/or chronic pelvic pain, which are the two major clinical symptoms (Wang *et al.*, 2020). Clinically, endometriosis is found in 21–47% of women with infertility and in 71–87% of women with chronic pelvic pain (Falcone and Flyckt, 2018).

Furthermore, the risk of malignant transformation in endometriosis, particularly ovarian endometriomas, is a clinical concern. A prospective cohort study has reported a 0.7% incidence of malignant transformation in ovarian endometriomas (Kobayashi *et al.*, 2007). Murakami *et al.* reported in two review articles that in endometriosis-associated ovarian cancer, the average time from the diagnosis of an endometriotic cyst to the diagnosis of ovarian cancer is 36 months, and clinically detectable cysts later diagnosed as ovarian cancer may have already contained cancer cells (Murakami *et al*., 2020a,b). Epidemiologically, endometriosis-associated ovarian cancer is reduced more by hysterectomy than by cystectomy alone, and genetic mutation analysis has identified oncogenic mutations in both endometriosis and normal endometrium, with the same mutations found in different endometriotic lesions. They also noted that most genetic mutations found in endometriosis originated in the normal endometrium (Murakami *et al.*, 2020a,b).

The aetiology of endometriosis is multifactorial. Various theories have been proposed to explain the ectopic presence of endometrial tissue, including retrograde menstruation, extrauterine stem cell theory, haematogenous or lymphatic dissemination of endometrial cells, coelomic metaplasia, and Müllerian duct rest theory. The implantation and progression of endometriosis are thought to involve immune dysregulation and multiple signalling pathways. Defects in immune surveillance within the peritoneal cavity create a microenvironment that favours immune evasion and adhesion of ectopic endometrial cells. Immune cells, such as neutrophils, macrophages, natural killer (NK) cells, and dendritic cells, play specific roles in angiogenesis, proliferation, and invasion of endometriotic cells. Additionally, cytokines and defensins secreted by these immune cells are implicated in the development of endometriosis (Abramiuk *et al.*, 2022; Reis *et al.*, 2024). Furthermore, several genetic loci associated with endometriosis risk have been identified (Riccio *et al.*, 2018; Wang *et al.*, 2020).

## PI3K/Akt/mTOR pathway activation in endometriosis

In studies using human specimens, mRNA analysis of tubal endometriosis samples revealed the activation of the mTOR pathway (Qi *et al.*, 2019). Activation of the PI3K/Akt/mTOR pathway has also been demonstrated using mRNA, PCR, and protein assays for uterine adenomyosis (Guo *et al.*, 2015a,b; Hu *et al.*, 2017; Xu *et al.*, 2018). At least six non-coding RNA molecules related to endometriosis and adenomyosis interact with upstream regulators of the mTOR pathway (Driva *et al.*, 2022). Elevated expression of the mTOR activators AXL and SHC1 has been observed in endometriosis (Honda *et al.*, 2008). Elevated expression of mTOR and Raptor have been observed in the peritoneal fluid of patients with endometriosis (Kim *et al.*, 2019b).

Activation of the mTOR pathway may be involved in the onset or progression of endometriosis through distinct mechanisms. Elevated PI3K expression, increased Akt phosphorylation, and reduced phosphatase and tensin homolog (PTEN) levels have been observed in ectopic and eutopic endometria of patients with endometriosis, especially in those with minimal to mild disease, highlighting the role of the PI3K/Akt/mTOR pathway in the onset of endometriosis (Madanes *et al.*, 2020). A meta-analysis of transcriptome microarrays reported that PI3K/mTOR pathway activation, transforming growth factor-β signalling, and interferon α/γ response are abundant only in stage III–IV endometriosis (Poli-Neto *et al.*, 2020). In this study, independent of the hormonal environment, an inflammatory profile predominates in stage I–II endometriosis, and macrophage polarization to the eutopic endometrium may be critical for disease progression. The high prevalence of NK T cells in the eutopic endometrium of women with endometriosis, regardless of the disease stage, suggests persistent stress or damage to eutopic endometria (Poli-Neto *et al.*, 2020).

The mTOR pathway is closely related to the endocrine system and has an activating effect that interacts with the oestrogen and progesterone pathways. Oestrogen receptors induce the expression of upstream regulators of the PI3K/Akt/mTOR pathway, such as receptor ligands, receptor tyrosine kinases, and signalling adaptors (Ciruelos Gil, 2014). Oestrogen-bound oestrogen receptors directly bind to the p85α regulatory subunit of PI3K and promote its phosphorylation (Hou *et al.*, 2014). Oestrogen stimulates the insulin-like growth factor (IGF) 1 receptor to induce PI3K signalling (Miller *et al.*, 2009). In contrast, the mTOR pathway enhances oestrogen receptor signalling. Specifically, mTORC1 facilitates the phosphorylation and activation of the oestrogen receptor via S6K1 (Alayev *et al.*, 2016). Activation of the PI3K/Akt/mTOR pathway may contribute to progesterone resistance (Driva *et al.*, 2022). The PI3K/Akt/mTOR pathway appears to regulate the response of ectopic endometrial tissue to progesterone and is probably associated with progesterone resistance, which is common in endometriosis (McKinnon *et al.*, 2018; Marquardt *et al.*, 2019; Li *et al.*, 2020). Activation of mTOR significantly upregulates the expression and secretion of stromal cell-derived factor 1, which is involved in oestrogen-mediated migration and recruitment of endothelial progenitor cells, thereby contributing to endometriotic lesions (Zhao *et al.*, 2020). Furthermore, the activation of mTOR signalling upregulates the expression of WD repeat domain protein 5, which interacts with the TET2 protein to induce the overexpression of oestrogen receptors, promoting the development of endometriosis (Xue *et al.*, 2021).

mTOR activation influences the development and progression of endometriosis by regulating autophagy and apoptosis factors, modulating adhesion and cell–cell adhesion molecules, promoting epithelial–mesenchymal transition angiogenesis, and controlling the microenvironment (Stubbings and Maund, 1988; McKinnon *et al.*, 2016; Jing *et al.*, 2019).

Inhibition of the mTOR signalling pathway has been shown to reduce the proliferation, migration, and invasion of endometrial stromal cells and promote autophagy and apoptosis in macrophages (Cao *et al.*, 2017; Liu *et al.*, 2020; Zhou *et al.*, 2021). Activation of vascular endothelial growth factor (VEGF) and hypoxia-inducible factor-1 has also been reported in adenomyosis (Goteri *et al.*, 2009).

**Figure 1.**
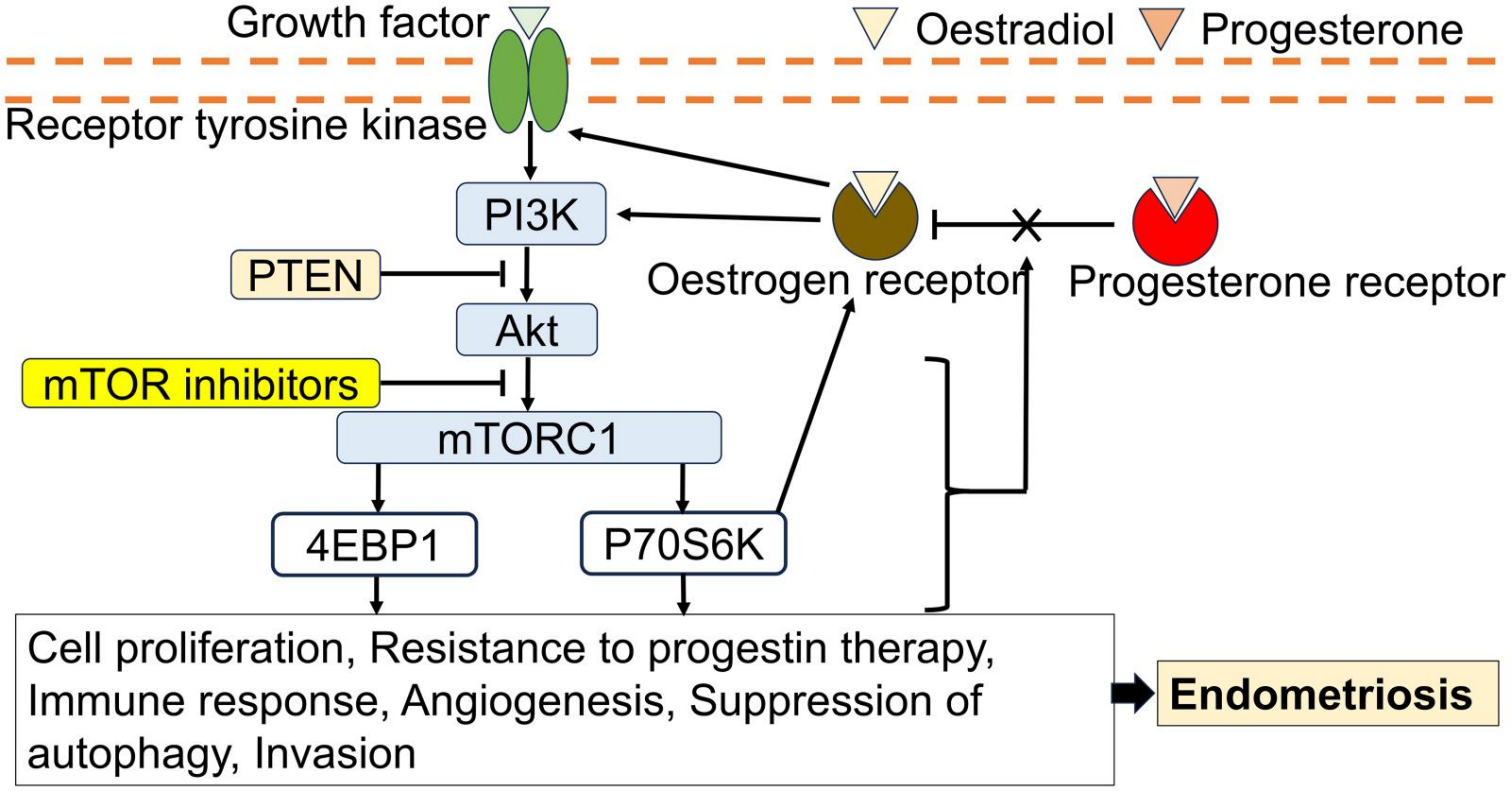
**The mTOR signalling pathway and the role of mTOR inhibitors in endometriosis.** PTEN, phosphatase and tensin homologue; mTOR, mammalian target of rapamycin; PI3K, phosphatidylinositol 3-kinase; Akt, protein kinase B; mTORC1, mammalian target of rapamycin Complex 1; 4EBP1, eukaryotic translation initiation factor 4E-binding protein 1; P70S6K, 70-kDa ribosomal protein S6 kinase.

Additionally, some copper metabolism-related genes, such as *PDHA1*, are downregulated during endometriosis, and mTOR is speculated to regulate copper metabolism in association with *PDHA1* (Wei *et al.*, 2023). The relationship between the activation of the mTOR pathway and endometriosis is shown in Fig. 1.

## PI3K/Akt/mTOR pathway activation and malignant transformation of endometriosis

The risk of malignant transformation in ovarian endometriotic lesions is associated with the PI3K/Akt/mTOR signalling pathway (Gadducci and Zannoni, 2020; Driva *et al.*, 2023). Whole-exome sequencing studies have shown that many PIK3 mutations found in normal uterine and endometriotic epithelial tissues are non-silent and frequently overlap with oncogenic mutations (Suda *et al.*, 2018). Additionally, the most common genetic alterations in ovarian clear cell carcinoma affect the V-Ki-ras2 Kirsten rat sarcoma viral oncogene homologue/PI3K pathway (Murakami *et al.*, 2017). In a clear cell or endometrioid carcinoma histotype, the AT-rich interactive domain-containing protein 1A (ARID1A), a tumour suppressor gene involved in chromatin remodelling, is often inactivated. This inactivation is associated with dysregulation of the PI3K/Akt pathway. Activation of the PI3K/Akt/mTOR pathway, combined with ARID1A mutations, is crucial for the malignant transformation of endometriotic lesions (Brush *et al.*, 1988; Samartzis *et al.*, 2013; Takeda *et al.*, 2016). Specific components of the mTOR complex, such as domain-containing mTOR-interacting protein, are similarly expressed in endometriotic and ovarian carcinoma tissues, highlighting the role of the mTOR signalling in connecting endometriosis to tumourigenesis (Rogers-Broadway *et al.*, 2019). Thus, although it is unclear whether mTOR inhibitors can prevent carcinogenesis, activation of the PI3K/mTOR pathway is likely involved in the malignant transformation of endometriosis.

## Therapeutic potential of PI3K/Akt/mTOR pathway inhibitors in endometriosis: preclinical insights

Preclinical studies have reported the efficacy of multiple PI3K/Akt/mTOR pathway inhibitors in the treatment of endometriosis. PI3K inhibitors have been shown to alleviate pain by inhibiting this signalling pathway in a rat model of sciatic endometriosis (Liu *et al.*, 2019b). The therapeutic effects of mTOR and aromatase inhibitors on follicle counts were examined in a rat model of endometriosis. Both drugs have shown therapeutic effects in endometriosis. However, although aromatase inhibitors reduce follicle counts, mTOR inhibitors preserve them (Kacan *et al.*, 2017). Additionally, in cultures of epithelial and stromal cells derived from human deep-infiltrating endometriosis and in a nude mouse transplantation model, cell proliferation was associated with increased endogenous oxidative stress and activation of the extracellular signal-regulated kinase (ERK) and mTOR/AKT pathways, and mTOR inhibitors demonstrated therapeutic effects (Leconte *et al.*, 2011).

mTOR inhibitors may be potential therapeutic agents for endometriosis because of their immunosuppressive properties. Endometriosis is recognized as an endocrine disorder, but it is also characterized by immune dysregulation, as evidenced by the activation of macrophages, NK cells, and T cells (Ahn *et al.*, 2015).

mTOR inhibitors have been shown to modulate cytokine production and regulate the activities of macrophages, NK cells, T cells, B cells, and antigen-presenting cells (Powell *et al.*, 2012; Weichhart *et al.*, 2015). However, the effects of mTOR inhibitors as immunosuppressants on endometriosis have not been thoroughly reported, even in preclinical studies, and further research is required.

A review article on the PI3K/AKT/mTOR pathway in angiogenesis demonstrated that activation of this pathway reduces VEGF secretion and angiogenesis (Karar and Maity, 2011). In fact, mTOR inhibitors also showed tumour-reducing effects in a mouse model of endometriosis, and VEGF regulation has been shown to be part of this effect (Ren *et al.*, 2016).

Endocrine therapy is the first choice of drug therapy for endometriosis. In research focused on non-endocrine therapies, it is important to understand their interactions with endocrine therapies. However, PI3K/Akt/mTOR pathway inhibitors may help improve resistance to endocrine therapy. Research on human endometriosis-derived stromal cells and a mouse endometriosis model showed activation of the Akt pathway in endometriosis and an inhibitory effect on proliferation of Akt inhibitors. It has been reported that progestin resistance is caused by activation of Akt, which can be improved by Akt inhibitors (Eaton *et al.*, 2013; Kim *et al.*, 2014).

Previous studies on non-hormonal drugs have suggested that their mechanisms of action include inhibition of the mTOR pathway. Similar to mTOR inhibitors, metformin has shown efficacy in clinical trials for endometrial cancer and is considered a promising treatment for endometriosis. The effects of metformin on endometrial cancer, endometriosis, and adenomyosis are believed to be mediated through inhibition of the mTOR pathway (Xue *et al.*, 2013; Kimber-Trojnar *et al.*, 2022). Similarly, statins, ginsenosides, and flavonoids have been reported as potential treatment candidates for endometriosis via inhibition of the mTOR pathway (Cao *et al.*, 2017; Kim *et al.*, 2019a; Zhang *et al.*, 2023a).

Additionally, it has been reported that part of the effect of hormonal agents on endometriosis may be mediated through the autophagy effect of mTOR inhibition (Choi *et al.*, 2015).

**Figure 2.**
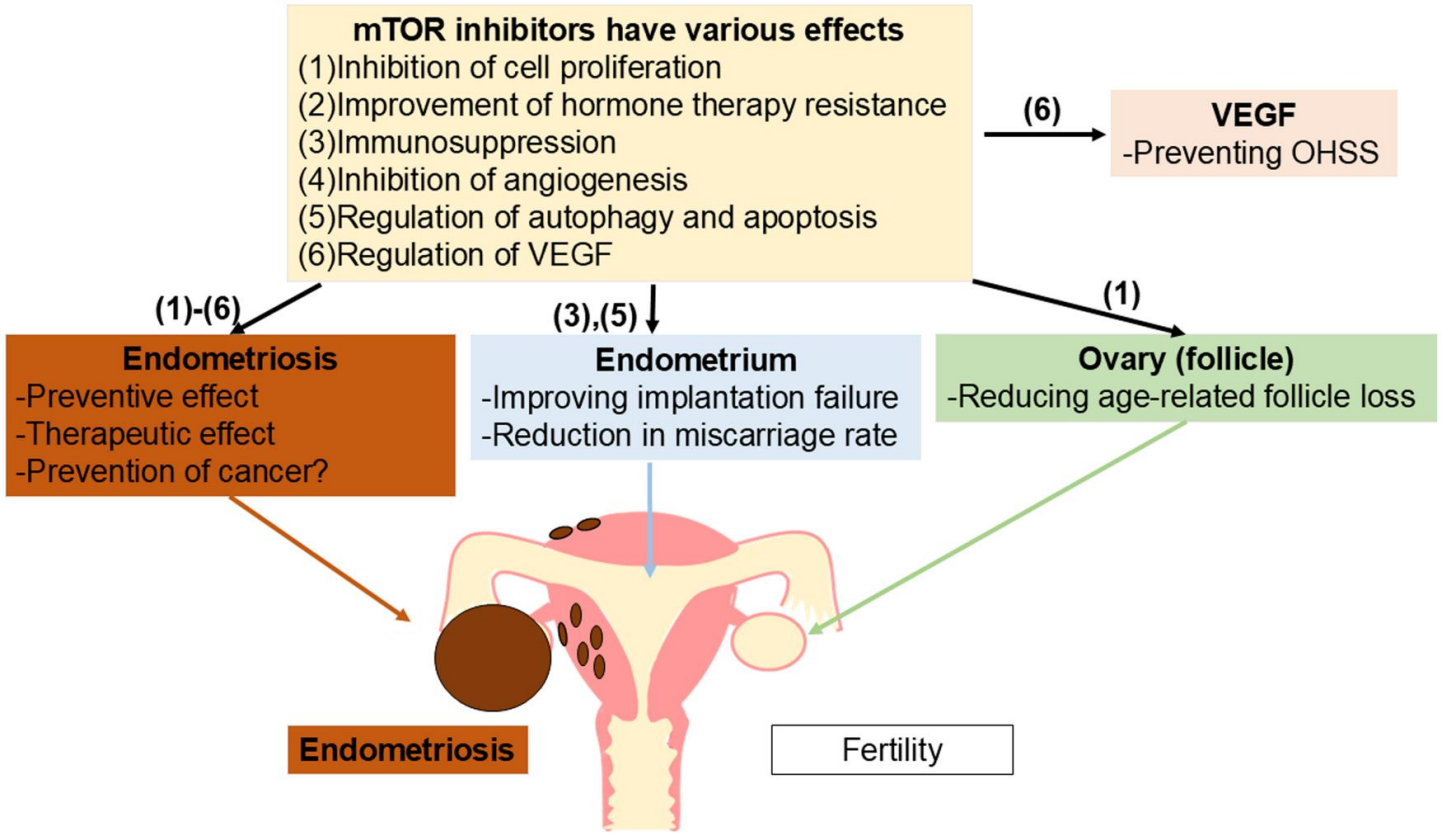
**Various effects of mTOR inhibitors on patients with endometriosis.** mTOR, mammalian target of rapamycin; VEGF, vascular endothelial growth factor; OHSS, ovarian hyperstimulation syndrome.

In summary, inhibitors of the PI3K/Akt/mTOR pathway may be potential treatments for endometriosis. However, all the papers cited are based on preclinical research, and further studies, including clinical trials, are needed (Fig. 2).

## Clinical trials and approval for PI3K/Akt/mTOR inhibitors

Currently, seven PI3K/Akt/mTOR pathway inhibitors are clinically available in Europe, including three mTOR, one Akt, and three PI3K inhibitors (Table 1). A notable characteristic of these inhibitors is that most are administered orally.

**Table 1.** List of PI3K/Akt/mTOR pathway inhibitors in clinical use.

	Agents	EMA-approved years	Route	Approved as immunosuppressant	Approved as antitumour agents
mTOR inhibitor	Rapamycin (sirolimus)	2001	Oral/DES^†^	Organ rejection Prevention of restenosis in coronary stents (DES^†^)	Lymphangioleiomyomatosis
mTOR inhibitor	Temsirolimus	2007	Injection		Renal cell carcinoma
mTOR inhibitor	Everolimus	2009	Oral/DES^†^	Organ rejection Prevention of restenosis in coronary stents (DES^†^)	Breast cancer (for hormone therapy resistance)^‡^ Renal cell carcinoma Neuroendocrine tumour TSC
PI3K inhibitor	Idelalisib	2014	Oral		Lymphoma (CLL, FL, SLL)
PI3K inhibitor	Copanlisib	2018	Injection		Lymphoma (FL)
PI3K inhibitor	Alpelisib	2020	Oral		Breast cancer (for hormone therapy resistance)^‡^
Akt inhibitor	Capivasertib	2024	Oral		Breast cancer (for hormone therapy resistance)^‡^

† Rapamycin and everolimus are used as drug-eluting stent-containing drugs.

‡ Approved in combination with endocrine therapy for hormone receptor-positive, human epidermal growth factor receptor 2-negative advanced or metastatic breast cancer that is resistant to endocrine therapy.

PI3K: phosphatidylinositol 3-kinase, Akt: protein kinase B, mTOR: mammalian target of rapamycin, CLL: chronic lymphocytic leukaemia, FL: follicular lymphoma, SLL: small lymphocytic lymphoma, TSC: tuberous sclerosis complex, DES: drug-eluting stent, EMA: European Medicines Agency.

As of 2024, there have been no reports of PI3K/Akt/mTOR pathway inhibitors being approved or tested in clinical trials, specifically for endometriosis. However, their demonstrated efficacy in combination with hormone therapy to improve hormone resistance in oestrogen-dependent cancers, such as breast and endometrial cancer, is of significant interest for future clinical research on endometriosis, which is also an oestrogen-dependent disease. Hormone-resistant breast cancer is one of the most common clinical indications for the use of PI3K/Akt/mTOR inhibitors. Similar to endometriosis, oestrogen plays a key role in the progression of hormone receptor-positive breast cancer, making endocrine therapy a cornerstone of treatment, even in postoperative adjuvant therapy or in cases of advanced recurrence. For instance, standard adjuvant therapy typically includes 5–10 years of endocrine treatment (tamoxifen or aromatase inhibitors, sometimes combined with GnRH analogues) (Davies *et al.*, 2013; Chen *et al.*, 2021). However, a subset of hormone receptor-positive breast cancers acquire resistance to hormone therapy and eventually relapse. Activation of the mTOR pathway contributes to this hormone resistance. PI3K/Akt/mTOR pathway inhibitors, such as everolimus (Yardley *et al.*, 2013), alpelisib (André *et al.*, 2019), and capivasertib (Turner *et al.*, 2023) have been approved for use in combination with oestrogen receptor antagonists or aromatase inhibitors to reintroduce hormone therapy in patients with recurrent or advanced hormone receptor-positive breast cancer.

Next, we discuss clinical research on PI3K/Akt/mTOR inhibitors in gynaecological cancers. Some recurrent advanced endometrial cancers are oestrogen-dependent, making hormone therapy a viable treatment option (Wagner and Backes, 2023). While clinical trials have demonstrated the efficacy of PI3K/Akt/mTOR inhibitors as monotherapy in endometrial cancer (Roncolato *et al.*, 2019; Avila *et al.*, 2022), as of 2024, the European Medicines Agency (EMA) has not granted approval for these inhibitors in endometrial cancer. However, the effectiveness of combining these inhibitors with endocrine therapy is noteworthy. Two phase II trials showed that everolimus, an mTOR inhibitor, combined with aromatase inhibitors, has significant efficacy (Slomovitz *et al.*, 2015, 2022). However, clinical research on these inhibitors in ovarian cancer remains limited (Avila *et al.*, 2022).

From an immunosuppressive perspective, mTOR inhibitors are currently the only inhibitors of the PI3K/Akt/mTOR pathway that are used as immunosuppressive agents. They are particularly utilized long-term following solid organ transplantation and have well-established safety profiles. Given that immune dysregulation plays a role in the onset and progression of endometriosis, the clinical use of mTOR inhibitors as immunosuppressive agents presents a compelling opportunity.

Examining the clinical history of PI3K/Akt/mTOR inhibitors, as shown in Table 1, all drugs with a long-term clinical track record of over 20 years were mTOR inhibitors. Recently, inhibitors targeting Akt and PI3K, which are upstream regulators of the mTOR pathway, have been introduced into clinical practice. Theoretically, these inhibitors can effectively target multiple signalling pathways, including mTOR signalling. However, it is essential to note that PI3K and Akt inhibitors are still in the early stages of clinical use. Generally, stronger inhibition of upstream components of signalling pathways can lead to more pronounced potential side effects. Given these factors, mTOR inhibitors, with their established safety profiles, may be the most suitable option for women desiring pregnancy and childbirth among PI3K/Akt/mTOR inhibitors.

Together, these findings suggest that while there are currently no reports of PI3K/Akt/mTOR pathway inhibitors being approved or tested in clinical trials for endometriosis, their efficacy in improving hormone resistance in oestrogen-dependent malignancies, their immunosuppressive properties, and their well-understood safety profiles from long-term use suggest that mTOR inhibitors may represent a safe therapeutic option for endometriosis.

## Additional benefits of mTOR inhibitors for patients with endometriosis desiring pregnancy

**Figure 3.**
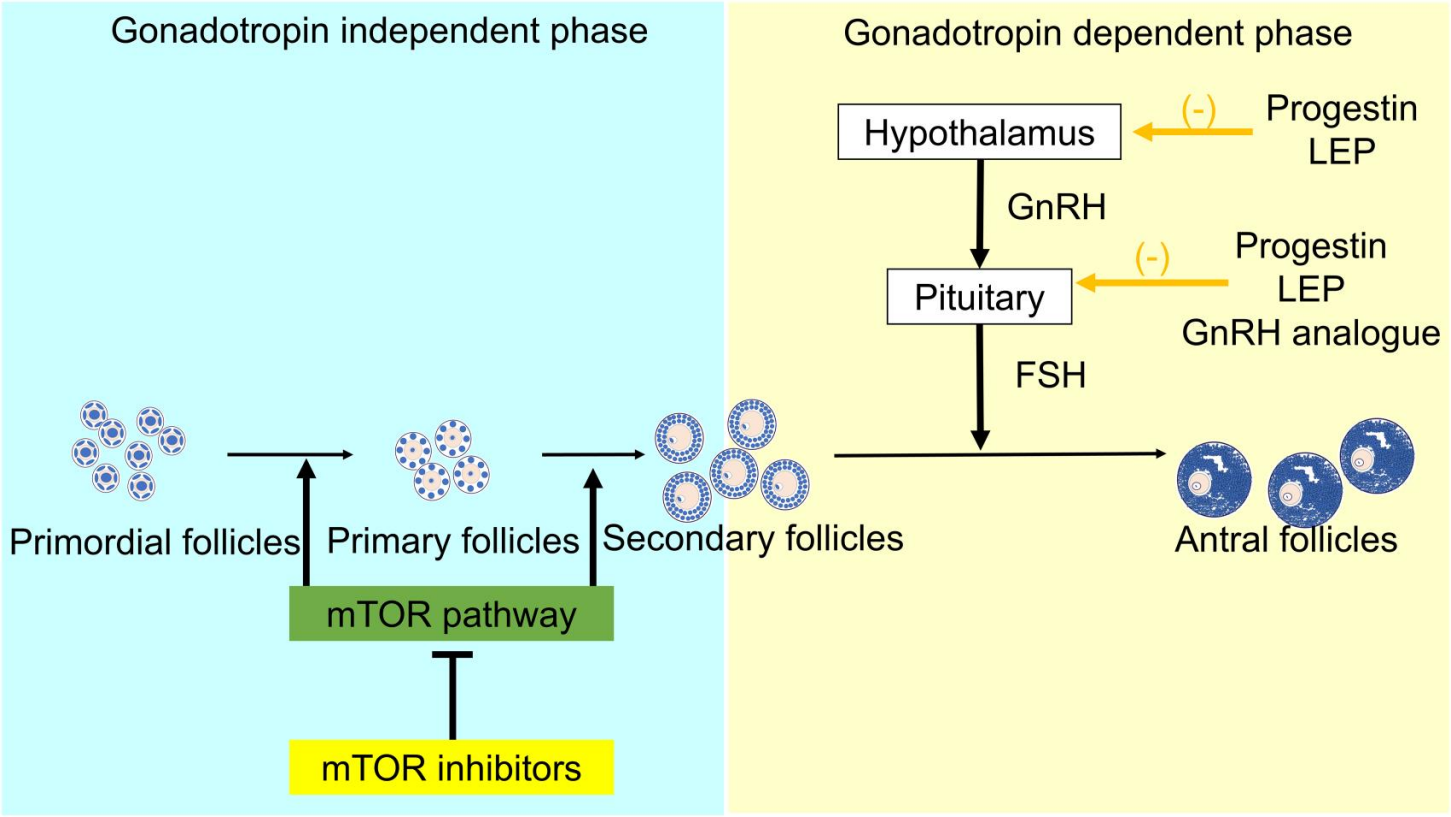
**Involvement of the mTOR pathway and gonadotropins in follicular development.** Follicular development can be divided into gonadotropin-independent and -dependent phases. The early stages, which are critical for preserving primordial follicles, occur during the gonadotropin-independent phase, which is primarily regulated by the mTOR pathway and not by hormones. mTOR, mammalian target of rapamycin; LEP, low-dose oestrogen–progestin combinations.

In *in vivo* studies, mTOR inhibitors have been reported to mitigate age-related follicular loss (Zhang *et al.*, 2013; Dou *et al.*, 2017; Yang *et al.*, 2018). A finite number of primordial follicles develop into primary, secondary, or antral follicles. The transition from primordial to primary follicles is gonadotropin-independent and is not regulated by hormonal agents but is primarily controlled by the mTOR pathway and anti-Müllerian hormone (Zhao *et al.*, 2021). This mechanism highlights the potential efficacy of mTOR inhibitors in preserving the ovarian reserve. Clinical reports that endometriosis causes ovarian dysfunction (Tan *et al.*, 2022) highlight the importance of reducing follicular loss (Fig. 3).

Activation of the mTOR pathway has been associated with spontaneous abortion and preterm birth through mechanisms involving autophagy and cyclooxygenase-2 signalling in the endometrium and uterine stromal cells. In *in vivo* studies, mTOR inhibitors may prevent these outcomes (Hirota *et al.*, 2011; Cha *et al.*, 2013; Lu *et al.*, 2021).

mTOR inhibitors also seem to offer benefits in managing ovarian stimulation during IVF embryo transfer cycles. A report on the effects of rapamycin, an mTOR inhibitor, in IVF for endometriosis-associated infertility involved 168 women who underwent two IVF cycles. Of these, 80 women were treated with rapamycin for 3 months before their second cycle. In the group receiving the mTOR inhibitor, oxidative stress markers decreased, antioxidant markers increased, and ageing markers (p16 and p21) decreased. Compared to the untreated group, the treated group required fewer days of stimulation, retrieved more oocytes, and had higher rates of fertilization, implantation, and clinical pregnancy. There are no reports of foetal abnormalities, and live birth rates were high (Fan *et al.*, 2024). Although this was not a randomized trial, the study suggests that short-term rapamycin treatment might improve IVF outcomes. Conversely, there is a case report of a woman taking mTOR inhibitors as immunosuppressants after heart transplantation who showed improved oocyte retrieval results after discontinuing the inhibitors before her second IVF cycle (Wald *et al.*, 2019). However, similar results have not been widely reported. *In vivo* studies have also suggested that mTOR inhibitors may prevent ovarian hyperstimulation syndrome (OHSS) by controlling immune responses and VEGF-mediated angiogenesis (Kosmas *et al.*, 2015; Kitsou *et al.*, 2016; Liu *et al.*, 2019a). mTOR inhibitors may also be beneficial during embryo transfer during IVF cycles. In a phase II trial involving patients with at least three prior implantation failures and elevated T helper 17 cell/regulatory T cell (Th17/Treg) ratios, 43 of 76 patients were treated with sirolimus, an mTOR inhibitor, while 33 remained untreated. In the mTOR inhibitor group, the Th17/Treg ratio decreased, and clinical pregnancy and live birth rates significantly increased (Ahmadi *et al.*, 2019). Implantation in patients with endometriosis has also been investigated in preclinical studies. In an *in vitro* study using endometrial stromal cells derived from patients with endometriosis, excessive activation of the PI3K/Akt pathway reduced the expression of IGF-binding protein-1, an implantation-specific gene, by decreasing nuclear forkhead box protein O1 levels. This effect was improved by the use of Akt inhibitors (Yin *et al.*, 2012) (Fig. 2).

In natural ovulation cycles, mTOR inhibitors cause mild and reversible menstrual irregularities, likely because of their inhibitory effects on follicular development (Sparagana *et al.*, 2017). However, numerous spontaneous pregnancies have been reported in women treated long-term with mTOR inhibitors after solid organ transplantation (Framarino-dei-Malatesta *et al.*, 2013). When using mTOR inhibitors in women aiming to conceive, it is essential to weigh the above benefits and drawbacks according to individual patient circumstances to determine the appropriateness of treatment.

## Challenges with hormonal treatment and potential of mTOR inhibitors in treating endometriosis

The primary pharmacological treatments for endometriosis are hormonal agents, including GnRH agonists (GnRHa), low-dose oestrogen–progestin combinations (LEP), and dienogest (DNG). Although the evidence supporting these hormonal therapies is robust, various issues remain unresolved. Therefore, progestin resistance remains a major concern. Additionally, while hormonal agents typically suppress ovulation, they do not prevent age-related loss of primordial follicles. The most critical limitation is that patients undergoing hormonal therapies are unable to become pregnant because ovulation is suppressed. Hormonal agents have been associated with specific adverse effects. However, GnRHa are associated with hypoestrogenism, which results in menopausal symptoms and limited long-term use. LEP must be administered cautiously to patients with obesity, those who smoke, and older individuals because of the increased risk of thrombosis. DNG can cause irregular bleeding and hypoestrogenic symptoms.

In contrast, mTOR inhibitors have various potential benefits in the treatment of endometriosis. They can mitigate hormone resistance when combined with hormonal agents, particularly progestins, and prevent age-related loss of primordial follicles. As monotherapy, mTOR inhibitors have the notable advantage of being compatible with pregnancy, allowing patients to conceive while undergoing treatment. However, the primary drawback of mTOR inhibitors is the lack of clinical trial data on endometriosis. Furthermore, managing side effects, such as metabolic disturbances, remains a critical consideration (Table 2).

**Table 2.** Characteristics of hormonal treatments for endometriosis and potential benefits of mTOR inhibitors.

	Hormonal agents	mTOR inhibitors
**Agents**	−GnRH analogue	−Rapamycin (sirolimus)
	−LEP	−Everolimus
	−DNG	
	−IUD	
**Major drawbacks**	−Progestin resistance	−Lack of clinical evidence in humans
	−No conception during treatment	
**Adverse events**	−GnRHa: menopausal symptoms, not for long-term use	−Stomatitis, hyperlipidaemia, hyperglycaemia, rash, infections^†^
	−LEP: Caution with obesity, smoking, age; thrombosis risk	
	−DNG: Irregular bleeding, hypoestrogenism symptoms	
**Other benefits**	−Controlling menstrual bleeding	−Mitigation of progestin resistance
	−Treatment effects on dysmenorrhoea	−Prevention of ageing-induced follicle loss
		−Compatible with pregnancy
		−Improvement of outcomes in ART

† Most adverse events are dose-dependent.

mTOR: mammalian target of rapamycin, GnRHa: GnRH agonists or antagonists, LEP: low-dose oestrogen–progestin combinations, DNG: dienogest, IUD: intrauterine device.

## Feasibility of controlling adverse events with mTOR inhibitors for treating endometriosis

When discussing the feasibility of mTOR inhibitors for the treatment of endometriosis, the most critical factor is the control of adverse events. In clinical use, mTOR inhibitors serve as antitumour or immunosuppressant agents. Common adverse events associated with mTOR inhibitors include stomatitis, glucose and lipid metabolism abnormalities, and rash. These adverse events are mostly mild, graded as Common Terminology Criteria for Adverse Events grade 2 or lower, and rarely lead to drug discontinuation when used as anticancer agents (Peterson, 2013). However, it is important to remember that endometriosis is a benign disease. Unlike for patients with cancer, treatments that have a high antitumour effect but also carry a high risk of adverse events are not acceptable in cases of endometriosis.

An important point to consider is that many adverse events associated with mTOR inhibitors are dose-dependent. Preclinical studies have shown that mTOR inhibitors are effective against endometriosis, even at relatively low concentrations (Table 3). This suggests that mTOR inhibitors may be effective at doses lower than those currently used in clinical applications, which is crucial for controlling the dose-dependent adverse effects of mTOR inhibitors. This could play a positive role in reducing the financial burden on patients.

**Table 3.** Comparison of doses of mTOR inhibitors for endometriosis or endometrial proliferation in *in-vivo* models with doses for antitumour or immunosuppressive effects.

Model	Disease	Dose	Doses aimed at antitumour or immunosuppressive effects in the same model
*In vivo* (mice), Temsirolimus (Leconte *et al.*, 2011)	Endometriosis	3 mg/kg/daily	10–25 mg/kg (Mattar *et al.*, 1990; Okui *et al.*, 2010; Vazakidou *et al.*, 2015; Chang *et al.*, 2018; Leiting *et al.*, 2023; Li *et al.*, 2023)
*In vivo* (mice), Everolimus (Erdemoglu *et al.*, 2009)	Endometrial proliferation	1.5 mg/kg/daily	2.5–12 mg/kg (Bradshaw-Pierce *et al.*, 2013)
*In vivo* (mice), Rapamycin (Ren *et al.*, 2016)	Endometriosis	<2 mg/kg/daily	5 mg/kg (Zeng *et al.*, 2010; Weng *et al.*, 2014; Zhou *et al.*, 2016)
*In vivo* (rat), Everolimus (Kacan *et al.*, 2017)	Endometriosis	1.5 mg/kg/daily	2.5–10 mg/kg (O’Reilly *et al.*, 2010; Gottschalk *et al.*, 2011; Piguet *et al.*, 2011; Bohra *et al.*, 2012; Pinto *et al.*, 2016)

mTOR: mammalian target of rapamycin.

The safety and efficacy of low-dose mTOR inhibitors have been the subject of extensive investigation within the field of anti-ageing research. Since the 2000s, when it was reported that mTOR inhibitors extended the lifespan of mice and fruit flies, a number of clinical studies have been conducted with the aim of extending the healthy lifespan of humans (Blagosklonny, 2019). A number of randomized controlled trials have demonstrated the anti-ageing effects of low-dose mTOR inhibitors on the immune and cardiovascular systems of healthy older adults. Notably, in many of these trials, low doses of mTOR inhibitors (<2 mg/day of rapamycin) were employed. In these trials, the incidence of adverse events associated with mTOR inhibitors was found to be comparable to that observed in the placebo group (Mannick *et al.*, 2021) or limited to mild and manageable side effects (Lee *et al.*, 2024). Moreover, a survey of adults using rapamycin off-label for the purposes of health, longevity, and anti-ageing revealed no notable adverse events (the most commonly used dose was approximately half the clinical dose) (Lee *et al.*, 2024).

## Teratogenicity of mTOR inhibitors

One of the primary concerns regarding the administration of mTOR inhibitors before and during pregnancy is teratogenicity. A case series of pregnant women who received mTOR inhibitors after organ transplantation generally reported favourable outcomes (Framarino-dei-Malatesta *et al.*, 2013). In the tuberous sclerosis complex, which is inherited in 50% of cases, foetal tumours may develop because of activation of the mTOR pathway. Notably, sirolimus is currently being clinically administered to pregnant women for the treatment of foetal cardiac rhabdomyoma caused by tuberous sclerosis complex (Will *et al.*, 2023). However, the evidence is not robust, necessitating further large-scale studies to investigate the teratogenicity and safety of mTOR inhibitors used during or immediately before pregnancy.

## Management of perioperative mTOR inhibitor medications

Owing to the potential of mTOR inhibitors to delay wound healing, it is advisable to consider a preoperative discontinuation period of ∼1 week. However, in a case series of major surgeries involving mTOR inhibitors, their safety was confirmed (Schwarz *et al.*, 2014; Heble *et al.*, 2018), and it is believed that there is no need to postpone surgery when emergency surgery is required, such as for ruptured ovarian chocolate cysts. Regarding the postoperative period, a study of patients who had received solid organ transplants showed no safety issues with starting mTOR inhibitors immediately after surgery (Mabood Khalil *et al.*, 2022).

## Conclusions

mTOR inhibitors may be potential therapeutic agents for endometriosis as they inhibit cell proliferation, improve resistance to hormone therapy, and provide immunosuppression. The required concentration for treating endometriosis may be lower than the concentrations used for immunosuppressants and antitumour drugs, suggesting that the side effects could be more manageable. Additionally, mTOR inhibitors may offer potential benefits for patients desiring pregnancy, such as reducing age-related follicle loss by inhibiting follicular development, improving IVF-IVF–embryo transfer' perhaps, and preventing OHSS by controlling VEGF.

## Data Availability

No new data were generated or analysed for the publication of this article.
